# Incidence and mortality trends of melanoma in Croatia, 1988-2008

**DOI:** 10.3325/cmj.2012.53.135

**Published:** 2012-04

**Authors:** Jelena Barbarić, Ariana Znaor

**Affiliations:** 1 Koprivničko-križevačka County Medical Center, Križevci, Croatia; 2 Andrija Štampar School of Public Health, University of Zagreb School of Medicine, Zagreb, Croatia; 3 Croatian National Institute of Public Health, Zagreb, Croatia

## Abstract

**Aim:**

To analyze melanoma incidence and mortality trends in Croatia 1988-2008, compare them with the trends in other populations, and identify possible changes in the trends.

**Methods:**

Incidence data were obtained from the Croatian National Cancer Registry and the mortality data from the Croatian Bureau of Statistics. United Nations population estimates were used for calculating the age-specific rates. Age-standardized rates were calculated by the direct standardization method, using the world standard population as a reference. To estimate incidence and mortality trends, we performed joinpoint regression analysis.

**Results:**

A significantly increasing incidence trend, with estimated annual percent change (EAPC) of 5.9% for men and 5.6% for women, was observed over the whole 21-year period and no additional joinpoints were identified. The overall incidence increase between the first and the last five-year period was 149% for men and 130% for women. Significant increase in the mortality trend was observed, with EAPC of 3.0% for men and 2.4% for women. No joinpoints were identified. The overall increase in mortality between the first and the last five-year period was 45% for men and 50% for women.

**Conclusion:**

Melanoma rates in Croatia are steadily and markedly rising, with similar trends to those in the countries with lower/intermediate incidence. It is important to further investigate the more specific causes of the increasing trends, as well as to implement effective public policies targeting the melanoma burden.

Malignant melanoma of the skin is a cancer originating in melanocytes, the pigment-producing cells of the skin (1). Unlike basal and squamous cell cancers of the skin that are rarely fatal, its five-year relative survival in Europe is 83.1% (2). With 286 new cases and 118 yearly deaths in men and 275 new cases and 79 deaths in women in Croatia in 2008, melanoma represented 2.6% of male cancer incidence and 1.1% of cancer deaths in men, and 2.9% of female cancer incidence and 1.4% of cancer deaths in women (3).

The strongest environmental risk factor for malignant melanoma in white populations is exposure to UV light (4). Intermittent sun exposure, especially before the age of 10 is considered to be a stronger risk factor than a continuous exposure (5,6). Other risk factors for malignant melanoma include invasive melanoma of the skin in one or more first degree-relatives, history of primary invasive melanoma, more than a hundred banal melanocytic nevi, three or more clinically atypical (dysplastic) nevi, pale Caucasian skin (type 1 or 2), red or blond hair, history of one or more severe blistering sunburns, sunbed use (especially before the age of 30), and history of pesticide exposure (6).

Generally, melanoma incidence rates in Caucasian populations increase with proximity to the Equator (7). However, there are variations across Europe, with Switzerland, Denmark, Norway, Sweden, and the Netherlands having the highest rates (15-18/100 000 in men and 16-22/100 000 in women) and Central and Southeastern Europe countries the lowest (4.4/100 000 for men and 4.3/100 000 for women) (8). Until now, recent trends in melanoma incidence and mortality have been less studied in the Mediterranean and Eastern European populations. The aim of this study was to analyze the melanoma incidence and mortality trends in Croatia 1988-2008, compare them with the trends in other populations, and identify possible changes.

## Materials and methods

### Data sources

Incidence data for the period 1988-2008 were obtained from the Croatian National Cancer Registry. The Registry, founded in 1959, covers the whole Croatian population (approximately 4.4 million persons) and relies on mandatory cancer notifications from primary and secondary health care sources and death certificates from the Croatian Bureau of Statistics. The Registry contributed data to the last three volumes of the Cancer Incidence in Five Continents series (9-11). Melanoma was defined as ICD-9 code 172 and ICD-10 code C43 (12). The number of melanoma deaths was obtained from the WHO Mortality Database (13). For calculating age-specific rates, we used the UN population estimates (14).

### Statistical analysis

Age-standardized rates of melanoma incidence in Croatia were calculated by the direct standardization method, using the world standard population as a reference (15). To describe incidence and mortality trends by calendar period, we carried out joinpoint regression analysis using the Joinpoint Regression Software (16). The aim of the approach is to identify possible joinpoints where a significant change in the log-linear trend occurs. To obtain the estimated annual percent change (EAPC), a regression line was fitted to the natural logarithm of the rates using calendar year as the response (17). In describing trends, the terms “significant increase” or “significant decrease” signify that the slope of the trend was statistically significant (*P* < 0.05). For non-statistically significant trends (*P* > 0.05), we used the terms “stable” (EAPC between -0.5% and 0.5%), “non-statistically significant increase” (EAPC>0.5%), and “non-statistically significant decrease” (EAPC<-0.5%). All statistical tests were two sided.

## Results

**Figure 1:**
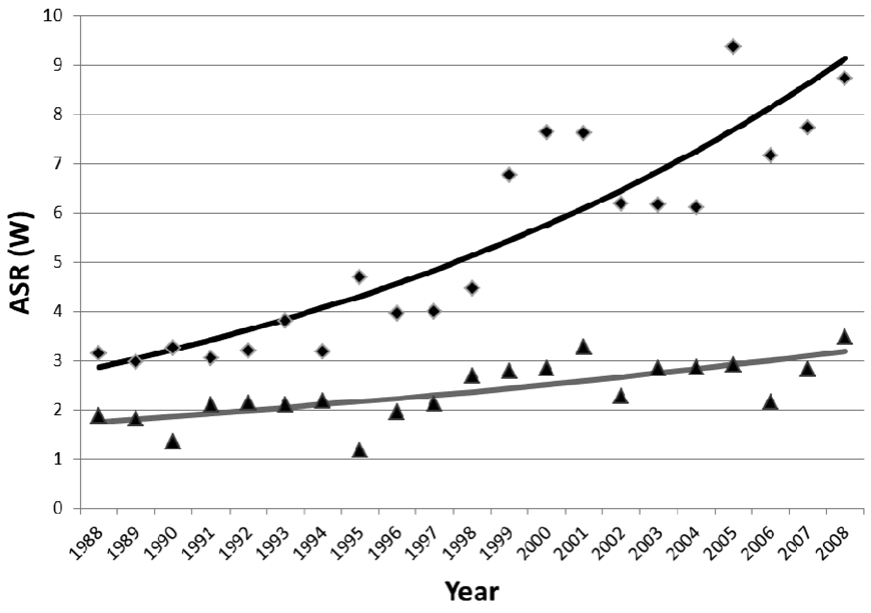
Joinpoint analysis for incidence and mortality of melanoma in Croatian men, 1988-2008. Rhombs – incidence; triangles – mortality; ASR(**W**) – age-standardized rates per 100 000 (using world standard population).

**Figure 2:**
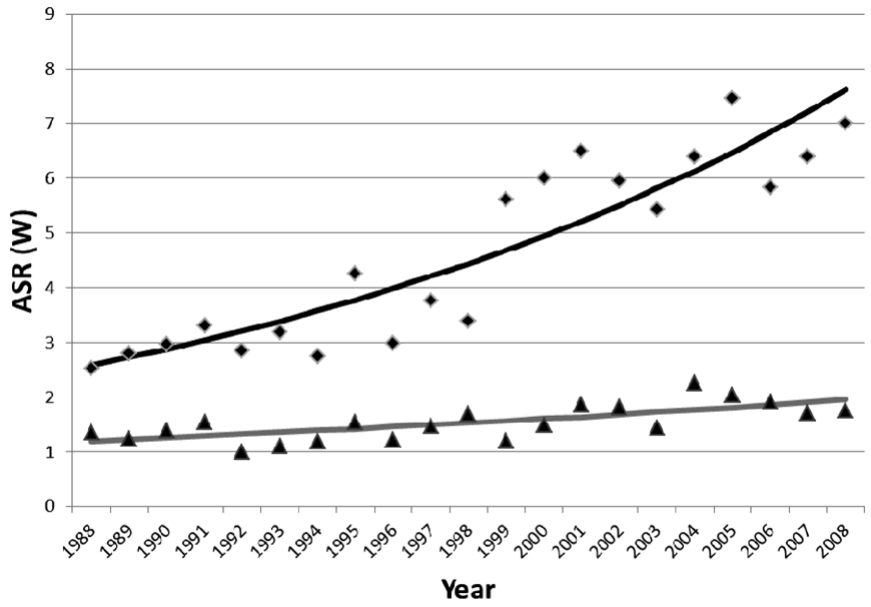
Joinpoint analysis for incidence and mortality of melanoma in Croatian women, 1988-2008. Rhombs – incidence; triangles – mortality; ASR(**W**) – age-standardized rates per 100 000 (using world standard population).

From 1988 to 2008, there were 3517 male and 3590 female melanoma cases. The number of new cases ranged from 81 to 303 annually for men, and from 87 to 284 for women (Table 1, 2). The significantly increasing incidence trend, with EAPC of 5.9% (95% confidence interval [CI], 4.5 to 7.5) for men and 5.6% for women (95% CI, 4.3 to 6.9) was observed over the whole 21-year period and no additional joinpoints were identified (Figure 1, 2). The overall incidence increase between the first and the last five-year period was 149% for men and 130% for women (Table 3). There were 1568 men and 1323 women who died of melanoma. The number of annual melanoma deaths ranged between 38 and 118 for men and between 39 and 94 for women (Tables 1, 2). Mortality trend showed a significant increase, with EAPC of 3.0% for men and 2.4% for women (95% CI, 1.6% to 4.4% and 1.3% to 3.6% respectively). No joinpoints were identified (Figures 1, 2). The overall increase in mortality between the first and the last five-year period was 45% for men and 50% for women (Table 3).

**Table 1:** Incidence and mortality of melanoma in Croatian men, 1988-2008

	Incidence			Mortality		
Year	N	crude rate	ASR(W)*	N	crude rate	ASR(W)
1988	81	3.7	3.1	51	2.4	1.9
1989	81	3.7	3.0	50	2.3	1.8
1990	88	4.0	3.3	38	1.7	1.4
1991	86	3.9	3.1	55	2.5	2.1
1992	91	4.1	3.2	60	2.7	2.1
1993	114	5.1	3.8	66	3.0	2.1
1994	97	4.3	3.2	67	3.0	2.2
1995	143	6.4	4.7	38	1.7	1.2
1996	122	5.4	4.0	61	2.7	2.0
1997	127	5.7	4.0	70	3.1	2.1
1998	140	6.3	4.5	84	3.8	2.7
1999	207	9.5	6.8	86	3.9	2.8
2000	240	11.1	7.6	91	4.2	2.9
2001	234	10.8	7.6	106	4.9	3.3
2002	194	9.0	6.2	71	3.3	2.3
2003	198	9.2	6.2	96	4.5	2.9
2004	191	8.9	6.1	92	4.3	2.9
2005	303	14.2	9.4	98	4.6	2.9
2006	239	11.2	7.2	73	3.4	2.2
2007	254	11.9	7.7	97	4.6	2.8
2008	287	13.5	8.7	118	5.6	3.5

*ASR(W) – age-standardized rates (using world standard population).

**Table 2:** Incidence and mortality of melanoma in Croatian women, 1988-2008

	Incidence			Mortality		
Year	N	crude rate	ASR(W)*	N	crude rate	ASR(W)
1988	87	3.8	2.5	53	2.3	1.4
1989	93	4.0	2.8	44	1.9	1.2
1990	106	4.6	3.0	50	2.1	1.4
1991	108	4.6	3.3	58	2.5	1.5
1992	100	4.2	2.9	39	1.6	1.0
1993	108	4.5	3.2	43	1.8	1.1
1994	102	4.2	2.8	45	1.9	1.2
1995	152	6.3	4.3	62	2.6	1.5
1996	106	4.4	3.0	44	1.8	1.2
1997	133	5.6	3.8	59	2.5	1.5
1998	119	5.0	3.4	65	2.7	1.7
1999	214	9.1	5.6	54	2.3	1.2
2000	218	9.3	6.0	65	2.8	1.5
2001	236	10.2	6.5	78	3.4	1.9
2002	212	9.2	6.0	71	3.1	1.8
2003	219	9.5	5.4	65	2.8	1.4
2004	238	10.3	6.4	94	4.1	2.3
2005	284	12.3	7.5	94	4.1	2.0
2006	235	10.2	5.8	81	3.5	1.9
2007	246	10.7	6.4	80	3.5	1.7
2008	274	12.0	7.0	79	3.5	1.8

*ASR(W) – age-standardized rates (using world standard population).

**Table 3:** Melanoma incidence and mortality in Croatia (1988-2002 and 2004-2008)

Period	Person – years*	Incidence				Mortality		
		N^†^	ASR(W)^‡^	absolute change (%)	N^§^		ASR (W)^‡^	absolute change (%)
**Men:**								
**1988-1992**	2.19	85	3.1	-	51		1.9	-
**2004-2008**	2.13	255	7.8	149	91		2.7	45
**Women:**								
**1988-1992**	2.34	99	2.9	-	49		1.3	-
**2004-2008**	2.30	255	6.6	130	86		1.9	50

*Mean annual male population expressed in million person-years at risk.

†Mean annual number of new cases.

‡Age-standardized rate (using world standard population).

§Mean annual number of deaths.

## Discussion

This study was the first to systematically analyze melanoma incidence and mortality trends in the Croatian population. Compared to other European countries from the GLOBOCAN 2008 database, Croatia had an intermediate melanoma incidence, with the ASR of 8.7/100 000 for men and 7.0/100 000 for women. However, with the ASR of 3.5/100 000 for men and 1.8/100 000 for women, it had the third highest male and fourth highest female mortality rate in Europe (8). As opposed to the majority of European countries, which had a higher incidence in women, Croatia had a higher incidence in men, similar to Australia and North America (8). Croatia also had a higher mortality rate in men.

Since the 1970s, melanoma incidence rates in populations of European origin doubled every 10 to 20 years (18). In Northern Europe, they became very high during the 1980s but have been leveling off since the mid-1990s (18,19). In Southern and Eastern Europe, on the other hand, the rates are still increasing (18,20-22). Mortality rates are leveling off in many populations with high incidence rates (such as Australia, USA, Scandinavian countries, the UK) (18,23-27), but are still increasing in most of Eastern and Southern European populations (18,21,22,27). Our results are similar to those in the countries with lower/intermediate incidence, eg, Italy with EAPC of 5.2% for men and 5.3% for women in the period 1985-2003 (22).

Croatian coast and islands have more than 2600 hours of sunshine per year, which contributes to intensive periodical sun exposure of the population (28). Since intermittent sun exposure is a major risk factor, the observed increases in Croatia may be attributed to popularization of tourism and holidaymaking in the 1950s and 1960s and the consumer practices of the 1970s and 1980s (29). In most countries, mortality rates have not increased as rapidly as incidence rates and it is controversial whether the increase in incidence is actually a consequence of increased surveillance and better detection of thin, minimally invasive melanomas (30). Due to the lack of information on stage and thickness of the tumors, it is impossible to determine whether increased awareness and early detection contributed to the increasing incidence trends in Croatia.

There has been only a modest progress in the management of advanced disease, and early diagnosis remains crucial in improving the survival (31,32). There are prevention and early detection strategies available, which have been developed and used in countries with higher incidence rates, including ABCDE guidelines and Glasgow 7-point checklist (33,34). Helping general public in recognizing melanomas and having them removed at an early stage was first done in the Queensland Melanoma Project (35), and then reproduced in the US and some European countries, such as the UK, resulting in earlier diagnosis and increase in the proportion of patients with thinner melanomas and better prognosis (34,36,37). Several population-based case-control studies suggest that whole body skin self-examination, as well as examination by clinicians, reduces the risk of thick melanomas (38,39). However, there are currently no completed randomized control trials that would confirm this. A randomized-controlled trial of a community-based melanoma screening program in Queensland, Australia, is expected to be complete in 2015 (34,40,41). A recent study in northern Germany has reported promising results in terms of feasibility and effectiveness of a systematic, two-step skin cancer screening program for a population of 1.88 million (42).

Croatia participates in Euromelanoma, pan-European prevention campaign against skin cancer that started in Belgium in 1999 (43-45). During Euromelanoma Week, the general public has an opportunity to have a free-of-charge dermatologist screening of the moles (46). It still remains to be seen whether this campaign will have an effect on the incidence and mortality trends in Croatia. Still, more effort should be put into increasing public awareness of melanoma and its prevention. Further research is needed to identify risk groups, their needs, and sun exposure-related behaviors to tailor Croatian prevention campaigns. Integration with the existing public-health activities should be considered, for example with the “Health Promoting Schools” project (45,46), to raise awareness of sun exposure risks and ways of protection among school children, their parents, and teachers.

Current high mortality rates clearly indicate a need to improve the outcomes of Croatian melanoma patients. At present, there is a great variation in the way they are diagnosed, treated, and followed. Policy-makers should ensure that every patient is treated to the same standards. As in other European countries, population aging is expected to occur, resulting in estimated 319 new male and 288 female melanoma cases in 2030, as well as 146 melanoma deaths in men and 88 in women (8). Therefore, an increased demand for melanoma-related medical services is expected, which will also require more detailed incidence and mortality data to enable adequate planning. What remains to be investigated are patterns of service provision and utilization, waiting times, survival patterns, as well as patient pathways, needs, and experiences.
